# Shared clinical and neural features of PTSD, dissociative disorders, and limbic epilepsy: a narrative review

**DOI:** 10.1016/j.ynstr.2026.100860

**Published:** 2026-10-23

**Authors:** Tatsushi Yokoyama, Yoshiharu Kim

**Affiliations:** a Laboratory of Optical Biomedical Science, Institute for Life and Medical Sciences, Kyoto University, Kyoto, 606-8507, Japan; b Emeritus Director General, National Institute of Mental Health, National Center of Neurology and Psychiatry, Tokyo, Japan

**Keywords:** Post-traumatic stress disorder (PTSD), Dissociative disorder, Limbic epilepsy, Engram cell, Ketamine-induced dissociation

## Abstract

The phenomenological closeness of post-traumatic stress disorder (PTSD) and dissociative disorders has been re-emphasized in recent diagnostic nomenclature, but their common neural pathogenesis is not yet clarified. Moreover, conventional neuroimaging studies have been observational, making it fundamentally difficult to establish causal relationships between abnormalities in specific brain regions and clinical symptoms. Here, we reviewed psychiatric symptoms in human limbic epilepsy, PTSD and dissociative disorders, together with findings from animal studies of limbic circuits, in order to explore causal links between limbic dysfunction and symptoms resembling those of PTSD and dissociative disorders. In human limbic epilepsy, epileptogenic activity arises within limbic-centered networks involving regions such as the hippocampus, amygdala and cingulate cortices, providing a clinical condition in which psychiatric symptoms can be linked to relatively identifiable neural disturbances. These symptoms overlap with key clinical domains of PTSD and dissociative disorders, including intrusive or altered memories, affective dysregulation, depersonalization/derealization, disturbances of consciousness and functional motor phenomena. This overlap raises the possibility that abnormal limbic activity may contribute to similar symptoms in PTSD and dissociative disorders. Animal studies further suggest that experimental manipulation of limbic structures and related circuits can produce PTSD-like and dissociation-like phenomena. Therefore, human limbic epilepsy and animal studies of limbic circuits complement observational neuroimaging studies by offering a framework for examining possible causal links between limbic dysfunction and PTSD- and dissociation-like symptoms.

## 1 Introduction

Among stress-related disorders, posttraumatic stress disorder (PTSD) and dissociative disorders are common and serious. Lifetime PTSD prevalence is approximately 3.9% and exceeds 15% in high-risk groups (Koenen et al., 2017). Internationally comparable estimates of the lifetime prevalence of dissociative disorders in the general population remain limited; however, a representative community study of 628 women in Sivas, Turkey, reported a lifetime prevalence of 18.3% (Şar et al., 2007). Yet treatments are limited: trauma-focused therapy is hard to disseminate, medications offer modest benefit, and evidence for treatments targeting dissociation is scarce. Clarifying their neural bases is crucial for new treatments.

Although PTSD and dissociative disorder diagnoses have increasingly converged, their neural substrates are seldom examined together. Neuroimaging has mapped limbic abnormalities in each, but it cannot determine whether these signatures cause symptoms, result from them, or are epiphenomenal. We therefore review limbic epilepsy as a “natural experiment,” where focal limbic events can generate experiences resembling core PTSD symptoms and dissociation. These observations raise the possibility that abnormal limbic activity may contribute to similar symptoms in PTSD and dissociative disorders. This hypothesis is further supported by recent findings in basic neuroscience regarding local circuits and molecular mechanisms underlying experimental fear circuitry.

As a narrative review, this article integrates representative findings from clinical psychiatry, epilepsy and basic neuroscience. Relevant English-language articles were identified through PubMed, Google Scholar and reference lists of key papers, covering literature available through September 2026, with priority given to studies directly relevant to the clinical phenomenology and neural mechanisms of the disorders discussed.

## 2 Symptomatology of PTSD and dissociative disorders

In recent years, the proximity between PTSD and dissociative disorders has been emphasized in the *Diagnostic and Statistical Manual of Mental Disorders* (DSM) (Stein et al., 2014). Below, we describe the symptoms of both conditions and their clinical overlap.

### 2.1 Symptomatology of PTSD

In DSM-5-TR (American Psychiatric Association, 2022), PTSD requires exposure to traumatic events (Criterion A) and symptoms across four clusters (Criteria B–E). Criterion B (intrusion symptoms) involves recurrent recollections of the traumatic event, which may be re-experienced in the form of flashbacks or nightmares. A flashback refers to a recollection in which a past traumatic experience is suddenly perceived as if it were occurring “here and now.” Criterion C (avoidance symptoms) reflects a tendency to avoid people, places, conversations, activities or other reminders associated with the trauma. Criterion D, termed negative alterations in cognition and mood (NACM), includes persistent negative beliefs about oneself or the world (e.g., “I am worthless,” “the world is dangerous”) and enduring emotions such as guilt, shame or fear. It also encompasses dissociative amnesia for important aspects of the traumatic event, markedly diminished interest or participation in significant activities, feelings of detachment or estrangement from others, and persistent inability to experience positive emotions. Criterion E (alterations in arousal and reactivity) includes symptoms such as irritability, hypervigilance, difficulty concentrating, exaggerated startle responses and sleep disturbance.

DSM-5 further specifies a dissociative subtype of PTSD, characterized by persistent or recurrent depersonalization and/or derealization in addition to the core PTSD clusters, often accompanied by emotional numbing and attenuated subjective experience.

### 2.2 Symptomatology of dissociative disorders

In DSM-5-TR, dissociation is a disruption or discontinuity in functions that are normally integrated, including consciousness, memory, identity, emotion, perception, body representation, motor control and behavior, and is considered to frequently arise because of traumatic experiences. Dissociative disorders are broadly classified into three major conditions.

In dissociative amnesia, important memories related to traumatic events are partially or completely lost and cannot be voluntarily recalled. In some cases, dissociative fugue may occur, in which individuals forget their past and begin a new life elsewhere. Depersonalization/derealization disorder is characterized by experiences of unreality, such as feeling detached from oneself (“I am not myself”) or perceiving the world as dreamlike, while reality testing remains intact. Dissociative identity disorder involves the presence of two or more distinct personality states that alternately take control of the individual, each with its own memories and emotional experiences.

### 2.3 Conceptual overlap between PTSD and dissociative disorders

As stated above, PTSD and dissociative disorders are both trauma-related conditions and share disruptions in integrative functions such as consciousness, emotion, memory and the sense of self. Consistent with this overlap, DSM-5/DSM-5-TR defines a dissociative subtype of PTSD, and several PTSD symptoms (e.g., flashbacks, amnesia, emotional numbing) can be conceptualized as dissociative phenomena. Although trauma exposure is not a formal diagnostic criterion for dissociative disorders, the DSM-5 notes its high prevalence among affected individuals, underscoring substantial construct overlap between the two conditions.

Presumably due to this historical background, the two conditions have often been investigated separately. Their conceptual convergence since DSM-5 raises the possibility of shared neural substrates and supports the value of analyzing and comparing them in parallel, but such attempts have so far been limited. Accordingly, we aim to elucidate neural mechanisms underlying both PTSD and dissociative disorders conceptually in the following sections.

## 3 Symptomatology of limbic epilepsy

Neuroimaging studies of PTSD and dissociative disorders report abnormalities in the limbic system—including the hippocampus, amygdala and cingulate cortex—as well as in the prefrontal cortices, thalamus and basal ganglia (Bromis et al., 2018; Hayes et al., 2012; Hinojosa et al., 2024; Roydeva and Reinders, 2021). However, observational neuroimaging studies fail to establish definitive causal links between brain findings and symptoms. This difficulty is overcome in epilepsy research, in which focal neuronal discharges are specified to generate certain symptoms. As discussed below, the link between epilepsy and PTSD or dissociation has often been discussed previously. We therefore examine its potential as a heuristic model for understanding PTSD and dissociative disorders.

Limbic epilepsy is epilepsy involving the limbic system—a network of brain regions, including the hippocampus, amygdala, and cingulate cortex, that integrates emotion, memory and autonomic function (Fig. 1). Interestingly, it shows substantial phenomenological overlap with PTSD and dissociative disorders. Clinically, limbic epilepsies are often described according to the predominant seizure-onset region, although the epileptogenic network may extend beyond that region.

**Fig. 1:**
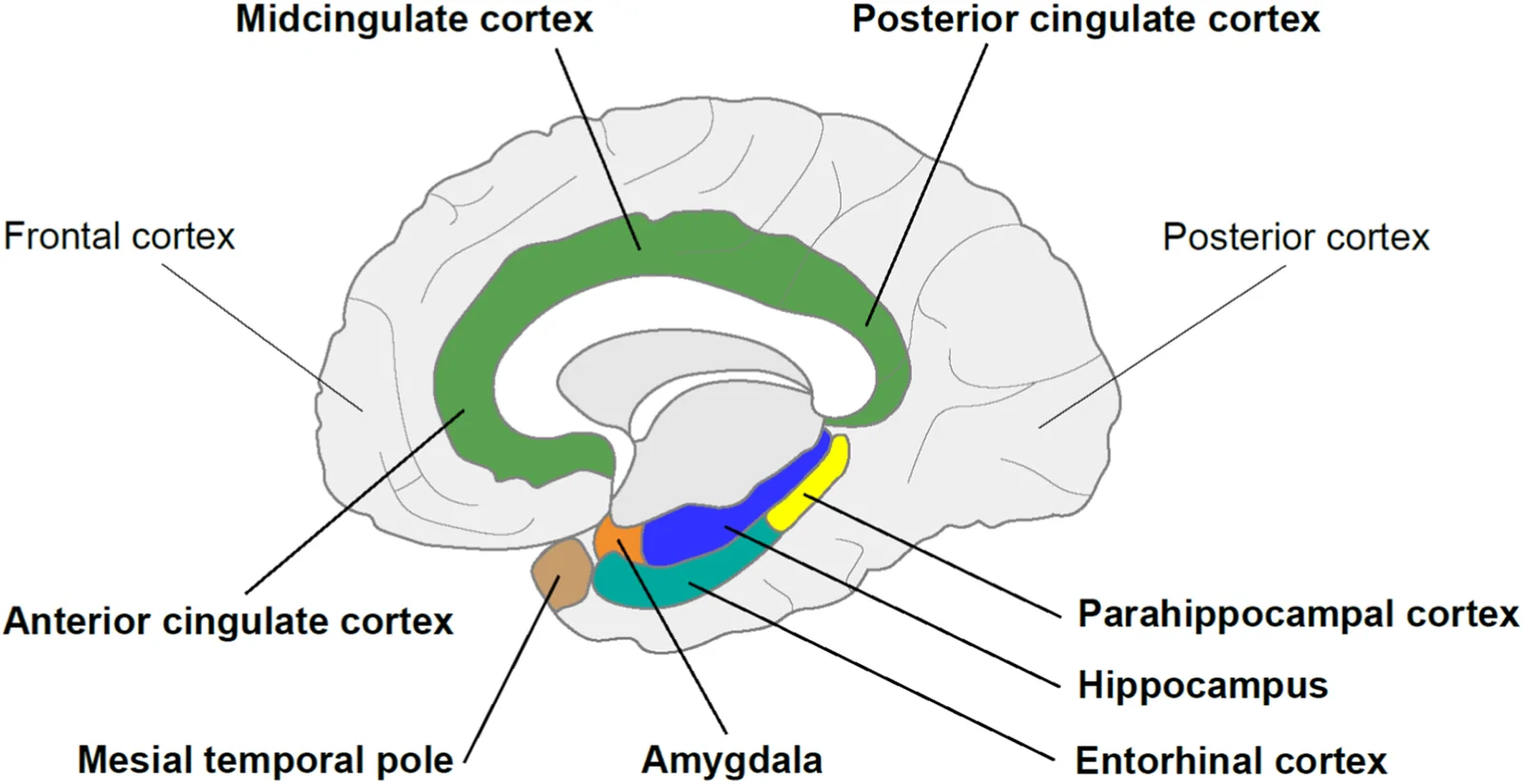
Limbic-centered network Schematic medial view of the human brain showing major limbic and related structures relevant to limbic epilepsy, including the anterior, midcingulate and posterior cingulate cortices, hippocampus, amygdala, parahippocampal cortex, entorhinal cortex and mesial temporal pole. The hippocampus and amygdala are located within the medial temporal lobe and are projected onto the medial surface for illustrative purposes.

### 3.1 Hippocampal epilepsy

Hippocampal epilepsy, a subtype of mesial temporal lobe epilepsy (mTLE), involves an epileptogenic focus in the hippocampus. Hippocampal sclerosis is the most common substrate of mTLE (Vinti et al., 2021), typically with onset in childhood–adolescence and links to febrile seizures, trauma, and post-encephalitic changes (Villamizar-Torres et al., 2024). MRI commonly shows hippocampal atrophy, and pathology shows neuronal loss and gliosis in CA1–CA3 and the dentate hilus (Villamizar-Torres et al., 2024).

In mTLE with hippocampal sclerosis, seizures most commonly begin with auras such as epigastric discomfort, fear or anxiety and autonomic symptoms (Villamizar-Torres et al., 2024). Patients also frequently experience depersonalization, déjà vu, jamais vu and affective changes, including dysphoric or euphoric feelings (Nayak and Bandyopadhyay, 2023; Villamizar-Torres et al., 2024). When consciousness becomes impaired during seizures, behavioral arrest and automatism may occur. Postictally, transient amnesia and confusion are often observed. There have also been reports of mnemonic experiences characterized by memories that emerge abruptly, in a fragmented manner without temporal continuity, and accompanied by a vivid sense of “being there” (Gloor et al., 1982; Kasper et al., 2010). Such mnemonic experiences are thought to arise primarily during seizures originating in the limbic system or following electrical stimulation of limbic structures (Gloor, 1990; Gloor et al., 1982), although reports by Penfield and colleagues have described similar phenomena induced by electrical stimulation of the lateral temporal cortex (Gloor, 1990).

### 3.2 Amygdalar epilepsy

Amygdalar epilepsy is a subtype of mTLE in which the epileptogenic focus is localized to the amygdala. mTLE with amygdala enlargement is considered a distinct entity from mTLE with hippocampal sclerosis, typically with adult onset and a lower frequency of febrile seizure history (Chakravarty et al., 2021). MRI often shows isolated unilateral amygdala enlargement. Reported histopathology includes focal cortical dysplasia, gliosis, and tumors/lesions such as ganglioglioma, oligodendroglioma, astrocytoma and hamartoma.

The ictal symptoms are generally similar to those observed in hippocampal sclerosis (Chakravarty et al., 2021). Although the presence of auras has been reported in more than 60% of cases, this proportion is lower than that seen in mTLE with hippocampal sclerosis. The most common auras include epigastric discomfort, déjà vu and fear. There are also reports indicating that interictal anxiety and depression are present in up to 90% of cases (Chakravarty et al., 2021).

### 3.3 Cingulate epilepsy

Cingulate epilepsy is a relatively rare form of focal epilepsy arising from the cingulate cortex and is characterized by heterogeneous semiology (Alkawadri et al., 2013; Inoyama and Devinsky, 2019; Powell et al., 2018). Reported pathology includes focal cortical dysplasia, gliosis, and astrocytoma. The cingulate cortex is anatomically divided into three regions—the anterior cingulate cortex (ACC), midcingulate cortex (MCC) and posterior cingulate cortex (PCC)—and seizure manifestations vary by the region involved.

In ACC epilepsy, auras occur in over half of patients and most often involve fear/anxiety or autonomic symptoms (e.g., palpitations, dyspnea) (Chassoux et al., 2025). Ictal features commonly include emotionally expressive facial changes, autonomic signs (sweating, flushing, heart-rate changes), vocalizations, and sudden complex/hypermotor behaviors; emotional vocalizations, laughter and ictal pouting (chapeau de gendarme) have been described. Loss of consciousness occurs in ∼35% and is not universal.

MCC epilepsy is dominated by hypermotor seizures, consistent with its connectivity to the supplementary motor area and motor cortices (Inoyama and Devinsky, 2019). Seizures may start with contralateral upper-limb clonic movements and evolve to brief tonic or dystonic posturing; gelastic and dacrystic seizures can reflect MCC-driven motor patterns (Inoyama and Devinsky, 2019).

PCC epilepsy often begins with subtle signs (brief behavioral arrest or mild impaired awareness) and later develops psychiatric/sensory symptoms such as paresthesia, fear and déjà vu, likely via propagation to limbic and other cortical regions (Inoyama and Devinsky, 2019). These manifestations are thought to reflect propagation of epileptic activity to the limbic system, other cingulate regions, and additional cortical areas. Semiology may resemble temporal lobe epilepsy, consistent with PCC connections to parahippocampal, entorhinal and hippocampal structures. Furthermore, dissociative symptoms have been reported as aura phenomena in seizures originating from the posteromedial cortex, including the posterior cingulate region, and similar dissociative experiences have been elicited by focal electrical stimulation of this area (Vesuna et al., 2020).

### 3.4 Network organization of limbic epilepsy

The considerable overlap in clinical manifestations among hippocampal, amygdalar, and cingulate epilepsies can likewise be understood from a network perspective. Recent studies have increasingly emphasized the network organization of temporal lobe epilepsy (Bartolomei et al., 2025; Feys et al., 2026). Mesial temporal seizures arise within a highly interconnected network comprising the hippocampus, amygdala, entorhinal cortex and the mesial temporal pole. The spatial extent of the epileptogenic network cannot be inferred directly from the location or size of a visible structural lesion, because there is little strict correspondence between the lesion and the epileptogenic zone. A structural lesion may constitute only one node within a broader epileptogenic zone network (EZN), and in some cases only part of the lesion itself may participate in the EZN. Moreover, the epileptogenic zone does not necessarily coincide with the symptomatogenic zone, defined as the region or network whose activation generates a particular clinical symptom, because ictal activity may propagate to other brain regions.

### 3.5 Limbic epilepsy as a sufficient causal basis for symptoms

Although the spatial extent of the epileptogenic network varies across individual cases, limbic epilepsy provides a useful basis for causal inference because abnormal epileptogenic activity can be directly linked to the emergence of clinical symptoms. Limbic epilepsy demonstrates that abnormal activity within a limbic-centered network can be sufficient to generate disturbances of emotion, memory, consciousness and self-experience, even though the symptomatogenic zone cannot always be localized.

## 4 Revisiting PTSD and dissociative disorders through the lens of limbic epilepsy

Although PTSD and dissociative disorders are traditionally classified as psychiatric conditions, several core symptoms resemble phenomena in limbic epilepsy, as compared below.

### 4.1 Comparative symptomatology of PTSD, dissociative disorders and limbic epilepsy

PTSD and dissociative disorders share multiple phenomenological features with limbic epilepsy (Table 1). First, the “re-experiencing” symptoms in PTSD involve a subjective experience in which past traumatic events are perceived as if they were occurring in the present; similar phenomena are recognized in limbic epilepsy as “mnemonic experiences”. In both conditions, these experiences are not reproduced in their original temporal sequence like the playback of a video but instead emerge abruptly as fragmented recollections lacking temporal continuity, with sensory or emotional elements suddenly coming to the forefront. Second, PTSD hyperarousal and negative alterations in cognition and mood (excluding emotional numbing) overlap with interictal mood disturbances described as interictal dysphoric disorder (IDD), including irritability, depressed mood, anergia, insomnia, atypical pain, anxiety, fear and euphoria (Blumer et al., 2004), although such disturbances are not unique to limbic epilepsy. Third, emotional numbing in PTSD and depersonalization/derealization in dissociative disorders resemble dissociative phenomena that may occur ictally in limbic epilepsy. Fourth, dissociative disorders include “automatic” phenomena accompanied by disruptions of consciousness, such as dissociative identity disorder and dissociative fugue. In this respect, they partially resemble automatisms in limbic epilepsy, in which consciousness is partially impaired, and behavior becomes automatic during seizures. This phenomenological overlap is only partial, as epileptic automatisms are typically brief and stereotyped, whereas alternate identity states and dissociative fugue are generally more prolonged and organized. Fifth, dissociative amnesia shares phenomenological features with amnestic phenomena in epilepsy, including ictal amnesia, postictal amnesia, and transient epileptic amnesia, in that both involve an inability to access previously available memories. This phenomenological overlap is partial, as dissociative amnesia typically involves impaired recall of important trauma-related memories, whereas epileptic amnesia occurs in temporal relation to seizures or, in transient epileptic amnesia, as recurrent brief episodes of amnesia (Baker et al., 2021). Taken together, these comparisons indicate phenomenological overlap across several symptom domains among PTSD, dissociative disorders, and limbic epilepsy, although the strength of correspondence varies across symptoms.

**Table 1:** Comparative symptomatology.

PTSD/Dissociative Disorders	Limbic Epilepsy	Strength
Re-experiencing symptoms	Mnemonic experiences	Close
Hyperarousal symptoms	Interictal dysphoric disorder	Partial
Avoidance symptoms	Learned interictal avoidance	Partial
Negative alterations in cognition and mood (excluding numbing)	Interictal dysphoric disorder	Partial
Numbing/depersonalization/derealization	Depersonalization/derealization	Close
Dissociative amnesia	Ictal/postictal amnesia; transient epileptic amnesia	Partial
Emergence of alternate personality	Automatisms	Partial

Avoidance is a core symptom of PTSD and has a counterpart in epilepsy as seizure-related avoidance, which may emerge as a learned interictal response. Although the two are not necessarily equivalent, this correspondence supports the interpretation of avoidance as a secondary behavioral response rather than a direct manifestation of limbic epileptic activity. Voluntary action and decision-making are likely mediated by brain regions outside the limbic system.

### 4.2 Co-occurrence of PTSD and epilepsy

PTSD is more prevalent in people with epilepsy than in the general population and has also been reported in association with mesial temporal lobe epilepsy (mTLE) (Ertan et al., 2025b; Pepi et al., 2024; Soncin et al., 2021, 2026). The traumatic and unpredictable nature of recurrent seizures, together with seizure-related fear learning, may contribute to the emergence or maintenance of PTSD symptoms, including hyperarousal, avoidance and re-experiencing (Soncin et al., 2021, 2026). In TLE, comorbid PTSD has also been associated with metabolic and functional connectivity alterations involving limbic and paralimbic regions (Soncin et al., 2022, 2024). Specifically, FDG-PET abnormalities involving the temporal and orbitofrontal cortices correlated with PTSD severity, while SEEG studies showed altered functional connectivity regarding the amygdala, hippocampus, insula and orbitofrontal cortex.

### 4.3 Bidirectional relationship between psychological trauma and epilepsy

The relationship may also be bidirectional. Psychological trauma before epilepsy onset was preferentially associated with temporal lobe epilepsy, with childhood trauma more frequent in this subgroup (Ertan et al., 2025a). Trauma after epilepsy onset was associated with a different psychiatric profile, including more postictal anxiety and mood symptoms (Ertan et al., 2025a). Experimental studies suggest that chronic stress induces glucocorticoid-related and BDNF-mediated changes in limbic circuits, which may lower seizure threshold and increase susceptibility to epileptogenesis (Becker et al., 2015; Jhaveri et al., 2023). This possibility has recently been conceptualized as “psychoepileptogenesis”, whereby psychological stress is proposed to prime vulnerable neural networks through stress-related neuroendocrine and neuroplastic changes, thereby lowering the threshold for subsequent epileptogenesis (Bartolomei et al., 2026). Together, these findings suggest the limbic system as a potential shared substrate.

### 4.4 Possibility of aberrant limbic activity in PTSD and dissociative disorders

Depersonalization and derealization during seizures have long been reported (Cassady and Baslet, 2023), and prior work has noted similarities between PTSD symptoms and complex focal seizures (Biran et al., 2020; Bob, 2015; Roca and Freeman, 2002). However, PTSD, dissociative disorders and limbic epilepsy have rarely been considered jointly from a phenomenological perspective.

As described in the preceding sections, evidence from limbic epilepsy indicates that aberrant limbic discharges can be sufficient to generate symptoms resembling those in PTSD and dissociative disorders. While PTSD and dissociative disorders lack the structural pathology typical of epilepsy, functional abnormalities including limbic regions have been consistently reported (Bromis et al., 2018; Hayes et al., 2012; Hinojosa et al., 2024; Roydeva and Reinders, 2021), although direct investigation at the level of neuronal activity remains limited. We therefore hypothesize that trauma-related plasticity in limbic circuits may increase susceptibility to transient aberrant firing, facilitating PTSD and dissociative symptoms.

In epilepsy and in intracranial electrical stimulation studies in humans, neural activity originating from different brain regions can produce similar symptoms. A possible explanation is that symptom-generating regions overlap across different forms of limbic epilepsy. Accordingly, a one-to-one correspondence between the epileptogenic focus and specific clinical symptoms is often difficult to establish, and it may be more appropriate to consider the strongly reciprocally connected limbic network as the relevant functional unit.

## 5 Local limbic circuits and molecular mechanisms

In the previous chapter, we linked abnormal limbic firing to disorder-specific symptoms. This chapter focuses on local circuit and molecular mechanisms, first revisiting the kindling model in epilepsy and PTSD and then reviewing advances beyond it.

### 5.1 Kindling model of epilepsy

In 1967, Goddard reported that repeated electrical stimulation of limbic structures—such as the amygdala, hippocampus and entorhinal cortex—in otherwise normal rats initially failed to induce convulsive seizures, but eventually led to the emergence of generalized seizures in response to stimulation, with a persistently lowered seizure threshold that was maintained for an extended period thereafter (Goddard, 1967). This phenomenon was interpreted as a pathological recruitment of physiological synaptic plasticity mechanisms and was subsequently termed the kindling model of epilepsy (Post, 2002). Once fully established, kindling is irreversible. Because standard rodent kindling does not generate spontaneous seizures, it is generally viewed as a model of secondary epileptogenesis and a “functional” form of temporal lobe epilepsy with minimal structural damage (Morimoto et al., 2004). Kindling also predicts antiseizure drug efficacy and remains widely used in preclinical screening. Although its relevance to human epilepsy is debated (Thompson, 2023), kindling remains an influential framework.

### 5.2 Kindling model of PTSD

The kindling model, originally developed in epilepsy, was later extended to PTSD. The PTSD-kindling hypothesis was explicitly linked to PTSD in a preliminary trial reporting symptom improvement with carbamazepine in combat veterans (Lipper et al., 1986). The PTSD-kindling hypothesis proposes that repeated stress induces limbic hyperexcitability, leading to cognitive and emotional dysregulation and potentially seizure-like activity within limbic circuits (Bob, 2015). Subsequently, it has been cited as a rationale for trials of antiseizure medications in PTSD.

The analogy rests on several observations. PTSD and dissociative disorders prominently involve limbic circuitry, which is also where kindling is readily induced in rodents (Goddard, 1967). Both PTSD/dissociation and rodent kindling typically show limited overt structural pathology. Moreover, stress-related reductions in somatostatin-positive inhibitory interneurons in the hippocampus and amygdala parallel findings reported after kindling stimulation (Rosen and Schulkin, 2022). In addition, kindled animals exhibit maladaptive fear-related behaviors—such as increased avoidance, fear and anxiety-like behaviors—even during interictal periods (Rosen and Schulkin, 2022), resembling interictal mood disturbances in epilepsy and symptoms of PTSD. Finally, the delayed emergence of seizures after repeated stimulation makes kindling a candidate framework for delayed-onset PTSD (Smid et al., 2022).

However, the kindling model of PTSD also has substantial limitations. While antiseizure drugs reliably suppress kindling and treat limbic epilepsy, evidence for their efficacy in PTSD remains insufficient despite a small early study (Lipper et al., 1986), and current guidelines do not support their efficacy for PTSD (American Psychological Association, 2025; National Institute for Health and Care Excellence, 2018). Evidence for antiseizure medications in dissociative disorders also remains limited, based on small studies of lamotrigine in depersonalization disorder (Sierra et al., 2003, 2006) and a systematic review of pharmacotherapy for dissociative disorders (Sutar and Sahu, 2019). Additional mechanistic proposals for PTSD include reduced adult hippocampal neurogenesis (Kida, 2019). These differences in treatment response suggest that, although abnormal neural excitability in relevant brain regions may be a shared phenomenon, the underlying local circuit and molecular mechanisms are likely to differ between epilepsy and PTSD. Furthermore, epileptiform EEG activity has not been consistently demonstrated in PTSD (Butt et al., 2019). However, conventional scalp EEG has limited sensitivity for detecting focal or deep limbic discharges (Casale et al., 2022), and the absence of scalp EEG abnormalities therefore does not necessarily exclude transient abnormal activity in deep limbic circuits. Finally, psychotherapy's efficacy in PTSD is difficult to reconcile with the presumed irreversibility of established kindling.

Taken together, although PTSD and rodent kindling converge on limbic circuitry, their underlying circuit and molecular mechanisms—and likely their patterns of aberrant neural activity—appear to differ. We therefore turn next to more recent animal-model approaches to further dissect the neural substrates of PTSD and dissociative disorders.

### 5.3 Animal models of PTSD: recent neuroscientific advances

Numerous animal models of PTSD have been developed (Verbitsky et al., 2020), including electric shock–based models, restraint stress models and predator stress models. Fear conditioning is also widely used to model fear-memory formation and retrieval: after pairing a neutral cue with an aversive stimulus, re-exposure to the cue elicits freezing, which can be quantified as an index of fear memory.

Recent advances in neuroscience allow PTSD-relevant fear-memory processes to be described in terms of limbic neuronal activity. In particular, the development of optogenetics has been useful. By genetically introducing light-sensitive channels into neurons and controlling neuronal firing with millisecond precision through light stimulation, optogenetics enables causal testing of the functional roles of small neuronal populations (Tye and Deisseroth, 2012). These methods can identify and manipulate neuronal ensembles known as “engram cells,” which are activated during memory formation and reactivated during subsequent recall. In fear memory studies, engram cells in the hippocampus or amygdala that were activated during fear conditioning can be selectively labeled and later reactivated using optogenetics, thereby inducing freezing behavior—representing fear memory recall—even in the absence of external stimuli (Tonegawa et al., 2015). Notably, the neurons activated in this process constitute only 5–20% of all neurons within a given brain region, demonstrating that the firing of a relatively small neuronal ensemble is sufficient for memory retrieval. Related work has shown that targeted, experimentally induced activation of a small number of CA1 place cells (∼15 responsive neurons on average) can bias memory-guided spatial behavior (Robinson et al., 2020). These findings demonstrate that activity in relatively small limbic neuronal ensembles can causally influence memory-related behavior.

### 5.4 Animal models of dissociative disorders: recent neuroscientific advances

Recent studies have modeled dissociation using dissociative NMDA receptor antagonists such as ketamine (Bera et al., 2025). In rodents, ketamine induces a characteristic 1–3 Hz slow oscillation in the retrosplenial cortex (RSC) (Vesuna et al., 2020), a region considered homologous to the human posteromedial cortex (PMC), including the posterior cingulate cortex (PCC) and anterior precuneus. Consistently, human intracranial EEG has identified a similar ∼3 Hz rhythm in the PMC during ketamine administration that correlates with subjective dissociative experiences (Tian et al., 2023). Comparable ∼3 Hz activity has also been observed in the same region during dissociative auras in epilepsy, suggesting a cross-species oscillatory substrate for dissociation (Vesuna et al., 2020). These findings support mechanism-based animal models of dissociation and motivate further work in this area. They also suggest that abnormal activity in the PCC and adjacent posteromedial limbic regions can be sufficient to induce dissociative symptoms, aligning with the central hypothesis in this review.

## 6 Conclusion

In this article, we compared PTSD and dissociative disorders with limbic epilepsy from a phenomenological perspective and examined their shared neural substrates. Whereas limbic epilepsy is characterized primarily by abnormal neuronal firing associated with structural pathology in regions such as the hippocampus and amygdala, PTSD and dissociative disorders may produce similar symptoms through trauma-induced plastic changes in neural circuits. The hypothesis that abnormal limbic activity may contribute to PTSD and dissociative symptoms is supported by findings from basic neuroscience.

### 6.1 Outstanding questions and perspectives

Several issues remain regarding the hypothesis that abnormal limbic activity contributes to PTSD and dissociative disorders. First, the nature of the putative aberrant limbic dynamics is unclear. While limbic epilepsy and kindling models implicate hyperexcitability and synchronized excitatory firing (Chen et al., 2020; Farrell et al., 2020), dissociative symptoms may reflect either excessive excitation or excessive inhibition within limbic circuits (Rosen and Schulkin, 2022). Indeed, although dissociation and depersonalization have been grouped within the same diagnostic category since DSM-III, the study of depersonalization has historically followed a distinct lineage with different theoretical traditions (Holmes et al., 2005; Sierra, 2009). Depersonalization may therefore represent a qualitatively distinct form of dissociation and is often accompanied by distress and anxiety, potentially placing it closer to anxiety disorders than to other dissociative conditions (Holmes et al., 2005; Hunter et al., 2003). Future work should test candidate mechanisms by integrating human neurophysiology with mechanistic animal studies.

Second, symptoms may depend on regions and networks beyond the limbic system. Although abnormal activity within a limbic-centered network may be sufficient to generate symptoms, non-limbic areas and large-scale networks may shape phenomenology; for example, depersonalization and derealization have been linked to different epileptic foci (Heydrich et al., 2019). One reason for such findings may be the difficulty, in human studies, of clearly distinguishing the epileptogenic zone from the symptomatogenic zone. Future research will therefore require parallel approaches combining interventional experiments in animal models with analyses of large-scale brain networks.

Taken together, PTSD and dissociative disorders may be viewed as predominantly non-lesional circuit disorders, in which trauma-related plasticity is hypothesized to increase susceptibility to transient abnormal limbic-network activity, whereas limbic epilepsies provide a comparison condition in which abnormal network activity is often associated with identifiable structural lesions. Framing PTSD and dissociative disorders as circuit disorders motivates limbic-network–guided interventions targeting network dynamics (e.g., neuromodulation and neurofeedback), while emerging single-cell and multi-omics approaches may link candidate firing patterns to molecular states, enabling a more precise mechanistic taxonomy and informing novel treatments.

## CRediT authorship contribution statement

**Tatsushi Yokoyama:** Writing – original draft, Funding acquisition, Writing – review & editing, Conceptualization. **Yoshiharu Kim:** Writing – review & editing, Conceptualization, Project administration.

## Funding

This work was supported by ACT-X-JST (JPMJAX253K to T.Y.), FOREST-JST (JPMJFR241Z to T.Y.), JSPS KAKENHI (JP25H02500, JP24K18240 to T.Y.).

## Declaration of competing interest

The authors declare that they have no known competing financial interests or personal relationships that could have appeared to influence the work reported in this paper.

## Data Availability

No data was used for the research described in the article.
